# Supplementary material for: The Prebiotic Kitchen: A Guide to Composing Prebiotic Soup Recipes to Test Origins of Life Hypotheses
Source: Life (Basel). 2021 Nov 11;11(11):1221. doi: 10.3390/life11111221 (PMC8618940; doi:10.3390/life11111221)
Supplement: Supplementary file 1 [file life-11-01221-s001.zip › life-1369279-Supplementary.pdf]

# Supplementary Materials: The Prebiotic Kitchen: A Guide to Composing Prebiotic Soup Recipes to Test Origins of Life Hypotheses

**Meteorite Assembled Mixture Design**

The meteorite recipe (Table S1) is based on published Murchison meteorite data. Any chemical that had a higher than 10 nmol/g reported concentration in the meteorite and were commercially available/affordable were used in the recipe. To generate the gram amounts per 100 g of material, the mole ratios were used to distribute the available chemicals over the weight percent of each chemical class. For example, amines make up 1.7 g per 100 g of meteoritic TOC. The only chemicals in abundance in this class were methylamine at 71 nmol/g and ethylamine at 18 nmol/g. Then, the nmol/g values were converted to ug/g using the molecular weight giving 2.2 μg/g of methylamine and 0.46 μg/g for ethylamine, which is 73% and 27% by weight, respectively. Therefore 1.25 g of methylamine and 0.46 g of ethylamine make up the entirety of the amines in the Murchison assembled soup. Using the provided spreadsheet, compounds can be added by changing the "include" value to "yes", which will shift all the gram amounts within that compound class without changing the combined amount of that compound class.

**Procedure for Preparing and Storing Meteorite Assembled Mixture**

To prepare the mixture, all of the water-soluble solids for 1 L were weighed, mixed in 500 mL of water, and divided into aliquots (e.g., 25 aliquots of 20 mL). Then, all of the water insoluble chemicals were mixed except the mineral oil, heated to mix, and divided into similar portions by weight (mass divided by 25). These mixtures were stored separately in the freezer. When ready to use, the insoluble components were combined with the aqueous components, ions were added in the desired concentrations, the pH was adjusted (typically using simple mineral acids and bases, e.g., HCl and NaOH), and water was added to get to desired final volume. Once prepared, the solution was stored at −20 °C. For long periods of time (more than one week), they were stored at −80 °C. Lyophilization was not advised, as some of the more volatile compounds may be lost in this process.

**Spark-Discharge Assembled Mixture Design**

The recipe for this mixture can be found in Table S3. To simplify the task of selecting compounds and setting their concentrations, we arbitrarily adjusted concentrations in multiples of 0.08 (0.08 mM for species detected in trace amounts, 0.16 mM, 0.24 mM, and up to 0.48 mM) and assigned a range of molar ratios to each concentration class (the ranges used can be found in Table S2). We chose 0.08 mM as it would allow us to avoid handling quantities that were too small to weigh out reliably. For the amino acid fraction, we set the concentration of glycine to 0.48 mM and used molar ratio data from the references listed in Table S2 to adjust the concentration values of the remaining compounds. We used glycine because its concentration is reported in many spark discharge experiment results and could be used to calibrate concentrations across the literature. While a racemic mixture of each amino acid would be preferable, we chose to include the isomer that is typically more readily available commercially (the L-form).

The concentrations of hydrogen cyanide, formaldehyde, and urea were taken from experiments done by Schlesinger and Miller [1] on a reducing gas mixture containing a 3:1 ratio of $CO_2$ and $CH_4$. As glycine concentrations were not reported in this study, we used HCN:glycine data from Ferus et al. [2] to set the concentrations. Nucleobase concentrations were selected based on findings reported by [2]. Note that guanine (0.08 mM) may or may not be included in the mixture based on the chosen pH, as it is insoluble at near-neutral pH values and above. Organic acid concentrations were adjusted based on data from early spark-discharge experiments [3], again using glycine as a benchmark. For all

compounds, if there were two or more values reported in the literature, we systematically chose the highest concentration.

**Procedure for Preparing and Storing Spark-Discharge Assembled Mixture**

To prepare the mixture, all solid compounds for 1 L of water were weighed and added to 500 mL of water. The liquids were added to the solution and all compounds were allowed to dissolve. The pH was adjusted (typically using simple acids and bases, e.g., HCl and NaOH) as needed. The volume of the solution was adjusted up to 1 L with water. The resulting solution was filter-sterilized using a 0.22-micron filter (nylon filters work well for this purpose as they are hydrophilic and low-binding). Then, they were separated into aliquots in pre-sterilized glass containers or vials. Once prepared, the solution was stored at −20 °C for short-term storage, or at −80 °C for longer periods. Alternatively, the aliquots were lyophilized or vacuum-dried and reconstituted with sterile water prior to use.

**Generic Synthesis Procedure for the Formose Reaction**

In total, 0.5 mL of formaldehyde (ASG reagent, 37% wt. in $H_2O$), 0.0126 g of glycolaldehyde (>97% purity), 4.5 mL of water (HPLC-grade or double distilled water), and 0.0705 g of calcium hydroxide (>96% purity) were placed in 10 mL borosilicate glass vial.

The reaction can be heated to speed it up or carried out in room temperature. The temperature selected will account for the total duration of the experiment (i.e., higher temperatures are allowed to react for shorter times), generally ranging from 30 minutes to 2 days. Priority was given to total reaction volume over concentration ranges, however, other researchers might choose to differ. The molarity of the reagents for this reaction is approximately is 1.3 M for formaldehyde and 0.4 M glycoaldehyde.

**Generic Synthesis Procedure for Formamide Condensation**

A total of 5 mL neat formamide (Reagent Plus®, >99.0 purity) was placed in a 10 mL borosilicate glass vial. The solution can be stirred using a magnetic stirrer in range 500–1200 rpm. It can be either UV irradiated or heated in the range of 50–160 °C [4]. The reaction is typically allowed to proceed for 48 hours to 1 week. Formamide is typically heated neat in such experiments, but other researchers might choose to add water, depending on the concentration range to be explored and overall objectives.

**Generic Synthesis Procedure for HCN Polymerization**

Equimolar 1 M (aqueous) solutions of sodium cyanide (NaCN) and ammonium chloride ($NH_4Cl$) were placed in a 25 mL glass vial, fitted with a silicon or teflon septa [5]. The pH was then adjusted to 9 with hydrochloric acid (HCl). To ensure anoxic conditions, the reaction was bubbled with nitrogen. Typically, the reaction was allowed to proceed for one month or longer (up to six months), at room temperature or heated at 38 °C [5].

## References


1. Schlesinger, G.; Miller, S.L. Prebiotic synthesis in atmospheres containing $CH_4$, CO, and $CO_2$. *J. Mol. Evol.* **1983**, *19*, 376–382, doi:10.1007/BF02101642.
2. Ferus, M.; Kubelík, P.; Knížek, A.; Pastorek, A.; Sutherland, J.; Civiš, S. High Energy Radical Chemistry Formation of HCN-rich Atmospheres on early Earth. *Sci. Rep.* **2017**, *7*, 1–9, doi:10.1038/s41598-017-06489-1.
3. Miller, S.L.; Urey, H.C. Organic Compound Synthesis on the Primitive Earth. *Science* **1959**, *130*, 7.
4. Saladino, R.; Botta, G.; Pino, S.; Costanzo, G.; Di Mauro, E. From the one-carbon amide formamide to RNA all the steps are prebiotically possible. *Biochimie* **2012**, *94*, 1451–1456, doi:https://doi.org/10.1016/j.biochi.2012.02.018.
5. Ruiz-Bermejo, M.; de la Fuente, J.L.; Marín-Yaseli, M.R. The influence of reaction conditions in aqueous HCN polymerization on the polymer thermal degradation properties. *J. Anal. Appl. Pyrolysis* **2017**, *124*, 103–112, doi:https://doi.org/10.1016/j.jaap.2017.02.015.
